# Supplementary material for: Niclosamide-loaded nanoparticles disrupt Candida biofilms and protect mice from mucosal candidiasis
Source: PLoS Biol. 2022 Aug 17;20(8):e3001762. doi: 10.1371/journal.pbio.3001762 (PMC9385045; doi:10.1371/journal.pbio.3001762)
Supplement: S1 Supporting information — (PDF) [file pbio.3001762.s001.pdf]

HITS: Ones in red are known antifungal drugs

antimycin A	inhibitor of cellular respiration, specifica
Chloroxin	has bacteriostatic, fungistatic, and ant
tioconazole	Antifungal
econazole	Antifungal
Miconazole	Antifungal
Clotrimazole	Antifungal
Isoconazole	Antifungal
**Niclosamide**	**antihelminthic drug**
terconazole	Antifungal
methiotepin maleate	has antipsychotic properties
voriconazole	Antifungal
ketoconazole	Antifungal
Auranofin	Anti-inflammatory
thonzonium bromide	monocationic detergent
thimerosal	antiseptic and antifungal agent
alexidine dihydrochloride	selective inhibitor of protein tyrosine pl
Fluorouracil	Antifungal
triclosan	antibacterial and antifungal agent
fluconazole	Antifungal
bifonazole	Antifungal
Hexachlorophene	disinfectant

Below are the corresponding OD of all the molecules that \
The deep orange ones were hits and labeled below

Prestwick Chemical Library plate 1

	No drug	No drug	3	4	5	6	7	8
A	0.177	0.168	0.152	0.154	0.174	0.156	0.165	0.175
B	0.169	0.130	0.132	0.129	0.134	0.127	0.150	0.140
C	0.146	0.118	0.103	0.093	0.096	0.096	0.102	0.099
D	0.135	0.095	0.086	0.090	0.090	0.091	0.096	0.100
E	0.163	0.115	0.105	0.087	0.094	0.098	0.110	0.097

F	0.149	0.097	0.079	0.083	0.083	0.092	0.108	0.091
G	0.154	0.109	0.102	0.090	0.086	0.092	0.087	0.096
H	0.145	0.098	0.096	0.091	0.097	0.095	0.098	0.094
I	0.146	0.123	0.114	0.100	0.097	0.109	0.115	0.104
J	0.152	0.117	0.116	0.100	0.115	0.107	0.107	0.100
K	0.138	0.114	0.115	0.095	0.107	0.114	0.087	0.085
L	0.143	0.093	0.090	0.091	0.084	0.083	0.089	0.088
M	0.150	0.139	0.119	0.115	0.091	0.093	0.093	0.093
N	0.153	0.123	0.115	0.093	**0.046**	0.085	0.097	0.088
O	0.152	0.128	0.115	0.107	0.086	**0.060**	0.101	0.099
P	0.160	0.140	0.128	0.104	0.111	0.097	0.094	0.089
					antimycin	Chloroxin		

Prestwick Chemical Library plate 2

	No drug	No drug	3	4	5	6	7
A	0.170	0.163	0.153	0.140	0.159	0.093	0.171
B	0.158	0.142	0.107	0.098	0.134	0.126	0.132
C	0.145	0.106	0.091	0.095	0.088	0.098	0.099
D	0.139	0.091	0.081	0.084	0.090	0.082	0.094
E	0.154	0.130	0.102	0.078	0.109	0.104	0.108
F	0.145	0.109	0.088	0.088	0.092	0.088	0.098
G	0.146	0.110	0.089	0.085	0.095	0.090	0.102
H	0.139	0.105	0.089	0.082	0.089	0.086	0.104
I	0.145	0.124	0.100	0.099	0.110	0.103	0.108
J	0.146	0.115	0.094	0.092	0.109	0.099	0.108
K	0.149	0.096	0.095	0.086	0.090	0.089	0.094
L	0.135	0.088	0.088	0.086	0.089	0.097	0.087
M	0.129	0.105	0.093	0.093	0.113	0.109	0.124
N	0.144	0.112	0.133	0.089	0.118	0.096	0.105
O	0.142	0.119	0.110	0.087	0.109	0.105	0.103
P	0.155	0.142	0.128	0.115	0.121	0.121	0.111

Prestwick Chemical Library plate 3

	No drug	no drug	3	4	5	6	7
A	0.175	0.168	0.155	0.156	0.170	0.158	0.173
B	0.158	0.129	0.112	0.120	0.137	0.146	0.130
C	0.144	0.103	0.101	0.113	0.102	0.109	0.113
D	0.126	0.090	**0.065**	0.095	0.103	0.104	0.104
E	0.162	0.111	0.105	0.094	0.116	0.123	0.114
F	0.147	0.106	0.100	0.087	0.097	0.106	0.114
G	0.141	0.106	0.102	0.099	0.110	0.100	0.112
H	0.144	0.108	0.088	0.087	0.096	0.100	0.103
I	0.149	0.124	0.114	0.102	0.105	0.123	0.115
J	0.155	0.116	0.100	0.116	0.111	0.117	0.124
K	0.149	0.107	0.102	0.101	0.107	0.106	0.097
L	0.136	0.104	0.085	0.088	0.086	0.085	0.092
M	0.140	0.117	0.109	0.100	0.098	0.103	0.101
N	0.142	0.141	0.106	0.103	0.100	0.098	0.109
O	0.145	0.120	0.103	0.101	0.098	0.110	0.107
P	0.158	0.145	0.125	0.118	0.109	0.107	0.116

Auranofin

Prestwick Chemical Library plate 4

	1	2	3	4	5	6	7
A	0.159	0.167	0.145	0.128	0.149	0.149	0.082
B	0.151	0.130	0.117	0.112	0.127	0.131	0.153
C	0.132	0.099	0.105	0.092	0.105	0.099	0.107
D	0.112	0.079	0.083	0.085	0.088	0.092	0.095
E	0.129	0.090	0.107	0.095	0.113	0.102	0.114
F	0.109	0.088	0.087	0.081	0.082	0.097	0.100
G	0.122	0.077	0.096	0.081	0.095	0.088	0.102
H	0.105	0.074	**0.068**	0.075	0.078	0.080	0.086
I	0.125	0.103	0.096	0.107	0.095	0.102	0.115

J	0.124	0.103	0.082	0.097	0.083	0.096	0.086
K	0.134	0.083	0.074	0.089	0.076	0.084	0.084
L	0.115	0.074	0.075	0.076	0.078	0.084	0.084
M	0.110	0.087	0.090	0.080	0.121	0.081	0.091
N	0.134	0.086	0.105	0.080	0.084	0.080	0.107
O	0.131	0.103	0.083	0.090	0.092	0.091	0.096
P	0.150	0.131	0.110	0.083	0.093	0.110	0.096

Fluorouracil

ally oxidative phosphorylation

iprotozoal properties

osphatases

were screened against OE in acetate media (384-well plate

9	10	11	12	13	14	15	16	17
0.167	0.160	0.148	0.147	0.151	0.141	0.137	0.142	0.144
0.119	0.127	0.142	0.144	0.122	0.152	0.116	0.131	0.136
0.109	0.110	0.118	0.111	0.106	0.109	0.108	0.086	0.103
0.089	0.102	0.098	0.107	0.122	0.103	0.098	0.098	0.100
0.115	0.106	0.117	0.117	0.113	0.119	0.108	0.106	0.114

0.104	0.102	0.101	0.102	0.094	0.097	0.102	**0.058**	**0.065**
0.103	0.091	0.109	0.088	0.101	0.095	0.099	0.105	0.101
0.099	0.097	0.098	0.088	0.098	0.095	0.092	0.099	0.103
0.106	**0.073**	0.096	0.095	0.108	0.104	0.096	**0.071**	0.102
0.114	0.103	0.102	0.108	0.105	0.115	0.113	0.097	0.106
0.097	0.095	0.095	0.128	0.117	0.087	0.094	0.098	0.102
0.088	0.079	0.092	0.085	0.096	0.099	0.077	0.089	0.090
0.103	0.090	0.101	0.097	0.097	0.084	**0.070**	0.081	0.138
0.085	**0.061**	0.080	0.089	0.091	0.083	0.086	0.098	0.092
0.097	0.102	0.116	0.093	0.099	0.091	0.098	0.100	0.101
0.101	0.099	0.097	0.112	0.097	0.091	0.100	0.096	0.098

tioconazole Miconazol Clotrimazole

econazole Isoconazole

8	9	10	11	12	13	14	15	16
0.173	0.156	0.171	0.155	0.157	0.153	0.165	0.156	0.179
0.146	0.122	0.129	0.136	0.128	0.125	0.128	0.114	0.100
0.106	0.105	0.109	0.110	0.117	0.103	0.115	0.109	0.109
0.093	0.101	0.099	**0.062**	0.095	0.102	0.095	0.103	0.098
0.110	0.105	0.104	0.105	0.121	0.101	0.098	0.101	0.109
0.110	0.098	0.108	0.102	**0.067**	0.115	0.104	0.100	0.108
0.100	0.099	0.093	0.100	0.097	0.106	0.104	0.110	0.104
0.093	0.095	0.097	0.095	0.094	0.103	0.097	0.094	0.103
0.114	0.095	0.091	0.113	0.104	0.106	0.112	0.097	0.104
0.109	0.104	0.088	0.116	0.106	0.120	0.116	0.110	0.106
0.106	0.098	0.102	**0.065**	0.091	0.096	0.103	0.098	0.096
0.094	0.079	0.094	0.098	0.097	0.093	0.095	0.094	0.098
0.096	0.095	0.094	0.123	0.097	0.082	0.103	0.095	0.097
0.103	0.100	0.111	0.100	0.093	0.094	0.096	0.100	0.092
0.120	0.105	0.096	0.102	0.103	0.097	0.097	0.091	0.099
0.120	0.113	0.108	0.103	0.108	0.108	0.099	0.094	0.098

terconazol voriconazole

methiotepin maleate

8	9	10	11	12	13	14	15	16
0.149	0.147	0.138	0.172	0.139	0.104	0.124	0.140	0.120
0.134	0.114	0.146	0.114	0.139	0.114	0.078	0.120	0.122
0.114	0.106	0.113	0.110	0.109	0.112	0.118	0.112	0.108
0.117	0.105	0.104	0.089	0.106	0.105	0.104	0.108	0.102
0.124	0.111	0.107	0.127	0.113	0.124	0.122	0.107	0.113
0.118	0.121	0.109	0.115	0.112	0.099	0.101	0.109	0.101
0.116	0.106	0.109	0.102	0.108	0.103	0.108	0.105	0.115
0.112	0.111	0.100	0.108	0.098	0.102	0.095	0.103	0.109
0.122	0.115	0.122	0.117	0.112	0.060	0.107	0.115	0.107
0.112	0.126	0.132	0.127	0.054	0.107	0.120	0.136	0.111
0.102	0.108	0.110	0.104	0.104	0.109	0.105	0.115	0.067
0.095	0.100	0.088	0.091	0.097	0.097	0.091	0.096	0.096
0.103	0.116	0.119	0.095	0.105	0.114	0.103	0.111	0.115
0.125	0.119	0.128	0.110	0.094	0.100	0.107	0.100	0.101
0.127	0.108	0.107	0.105	0.109	0.087	0.099	0.106	0.099
0.116	0.110	0.109	0.105	0.101	0.095	0.100	0.094	0.088
				thonzoniu	thimerosal			alexidine d

8	9	10	11	12	13	14	15	16
0.131	0.132	0.132	0.149	0.095	0.126	0.129	0.144	0.127
0.140	0.131	0.136	0.107	0.139	0.131	0.128	0.113	0.138
0.104	0.106	0.104	0.090	0.102	0.094	0.107	0.114	0.101
0.098	0.097	0.107	0.100	0.096	0.100	0.095	0.095	0.095
0.097	0.100	0.104	0.109	0.114	0.104	0.096	0.119	0.106
0.099	0.092	0.096	0.095	0.099	0.094	0.102	0.093	0.099
0.096	0.097	0.101	0.096	0.096	0.092	0.103	0.093	0.091
0.089	0.083	0.091	**0.066**	0.089	0.090	0.089	0.092	0.088
0.115	0.103	0.106	0.118	0.108	0.108	0.112	0.104	0.106

0.094	0.097	0.100	0.111	0.104	0.098	0.102	0.094	0.103
0.086	0.081	0.101	0.102	0.100	0.088	0.091	0.096	0.088
0.086	0.092	0.090	0.091	0.094	0.086	0.089	0.093	0.093
0.117	0.118	0.087	0.094	0.096	0.101	0.114	0.088	0.090
0.086	0.092	0.092	0.091	0.098	0.094	0.087	0.096	0.091
0.096	0.096	0.099	0.089	0.087	0.087	0.087	0.087	0.083
0.085	0.099	0.091	0.094	0.095	0.081	0.088	0.096	0.087

triclosan

es)

18	19	20	21	22	23	24
0.118	0.148	0.137	0.141	0.139	0.135	0.135
0.116	0.093	0.121	0.139	0.120	0.126	0.124
0.105	0.100	0.102	0.106	0.089	0.112	0.116
0.096	0.097	0.095	0.092	0.109	0.098	0.107
0.097	0.111	0.111	0.103	0.110	0.096	0.108

0.086	0.095	0.099	0.114	0.085	0.095	0.104
0.101	0.097	0.108	**0.061**	0.098	0.097	0.099
0.086	0.098	0.098	0.098	0.097	0.093	0.104
0.109	0.110	0.105	0.104	0.107	0.101	0.113
0.111	0.112	0.101	0.084	0.098	0.101	0.110
0.117	0.122	0.092	0.092	0.098	0.095	0.102
0.092	0.114	0.088	0.090	0.091	0.092	0.105
0.104	0.099	0.090	0.096	0.104	0.113	0.093
0.084	0.091	0.088	0.092	0.087	0.089	0.102
0.101	0.098	0.094	0.094	0.096	0.103	0.094
0.104	0.098	0.099	0.092	0.089	0.096	0.108

Niclosamide

17	18	19	20	21	22	23	24
0.164	0.128	0.162	0.151	0.149	0.114	0.138	0.157
0.137	0.127	0.114	0.119	0.116	0.126	0.140	0.139
0.105	0.110	0.104	0.102	0.107	0.112	0.110	0.120
0.075	0.099	0.095	0.099	0.101	0.101	0.100	0.112
0.098	0.102	0.110	0.114	0.109	0.113	0.112	0.127
0.108	0.108	0.092	0.094	0.107	0.107	0.099	0.119
0.121	0.093	0.111	0.105	0.108	0.100	0.100	0.109
0.099	0.104	0.101	0.104	0.102	0.096	0.099	0.107
0.101	0.097	0.106	0.104	0.082	0.110	0.103	0.114
0.114	0.092	0.104	0.109	0.105	0.109	0.105	0.110
0.095	0.100	0.091	0.102	0.095	0.105	0.102	0.112
0.092	0.095	0.095	0.099	0.094	0.108	0.108	0.115
0.080	0.097	**0.064**	0.105	0.100	0.116	0.098	0.112
0.100	0.090	0.100	0.096	0.094	0.085	0.096	0.109
0.096	0.092	0.080	0.093	0.095	0.077	0.099	0.098
0.095	0.088	0.084	0.089	0.081	0.089	0.094	0.106

ketoconazole

17	18	19	20	21	22	23	24
0.132	0.131	0.146	0.134	0.141	0.148	0.140	0.153
0.110	0.121	0.121	0.138	0.099	0.138	0.136	0.151
0.108	0.112	0.108	0.110	0.099	0.104	0.110	0.124
0.111	0.107	0.107	0.123	0.104	0.102	0.103	0.125
0.111	0.137	0.118	0.117	0.119	0.120	0.118	0.133
0.112	0.100	0.111	0.102	0.119	0.101	0.114	0.128
0.102	0.110	0.102	0.109	0.113	0.125	0.114	0.126
0.116	0.094	0.105	0.107	0.098	0.105	0.104	0.119
0.126	0.113	0.108	0.115	0.106	0.110	0.102	0.123
0.142	0.109	0.136	0.112	0.114	0.110	0.113	0.119
0.101	0.096	0.108	0.108	0.108	0.103	0.107	0.114
0.098	0.102	0.093	0.097	0.101	0.101	0.117	0.117
0.122	0.109	0.107	0.105	0.099	0.100	0.103	0.113
0.102	0.104	0.107	0.109	0.108	0.102	0.099	0.121
0.103	0.098	0.101	0.103	0.097	0.100	0.101	0.100
0.098	0.090	0.102	0.115	0.101	0.100	0.107	0.120

dihydrochloride

17	18	19	20	21	22	23	24
0.131	0.144	0.123	0.133	0.117	0.127	0.141	0.154
0.125	0.124	0.118	0.138	0.147	0.137	0.130	0.151
0.102	0.105	0.103	0.102	0.101	0.104	0.105	0.120
0.095	0.096	0.101	0.099	0.081	0.096	0.094	0.106
0.100	0.080	0.115	0.104	0.126	0.093	0.101	0.115
0.099	0.116	0.096	0.104	**0.062**	0.095	0.089	0.105
0.083	0.098	0.095	0.092	0.096	0.079	0.087	0.099
0.088	0.092	0.092	0.094	0.088	0.092	0.087	0.114
0.110	0.096	0.112	0.100	0.099	0.103	0.095	0.106

0.099	0.101	0.104	0.098	0.099	0.102	0.092	0.113
0.093	0.089	0.088	0.097	0.089	0.078	0.089	0.105
0.085	0.092	0.091	0.089	**0.067**	0.088	0.084	0.090
0.087	0.121	0.070	0.110	0.089	0.099	0.086	0.118
0.090	0.099	0.094	0.099	0.094	0.093	0.087	0.110
0.094	0.086	0.095	0.084	0.091	0.088	0.087	0.099
0.096	0.093	0.091	0.098	**0.057**	0.081	0.090	0.117

fluconazole

bifonazole

Hexachlorophene
