# Supplementary figures and images for: Niclosamide-loaded nanoparticles disrupt Candida biofilms and protect mice from mucosal candidiasis
Source: PLoS Biol. 2022 Aug 17;20(8):e3001762. doi: 10.1371/journal.pbio.3001762 (PMC9385045; doi:10.1371/journal.pbio.3001762)

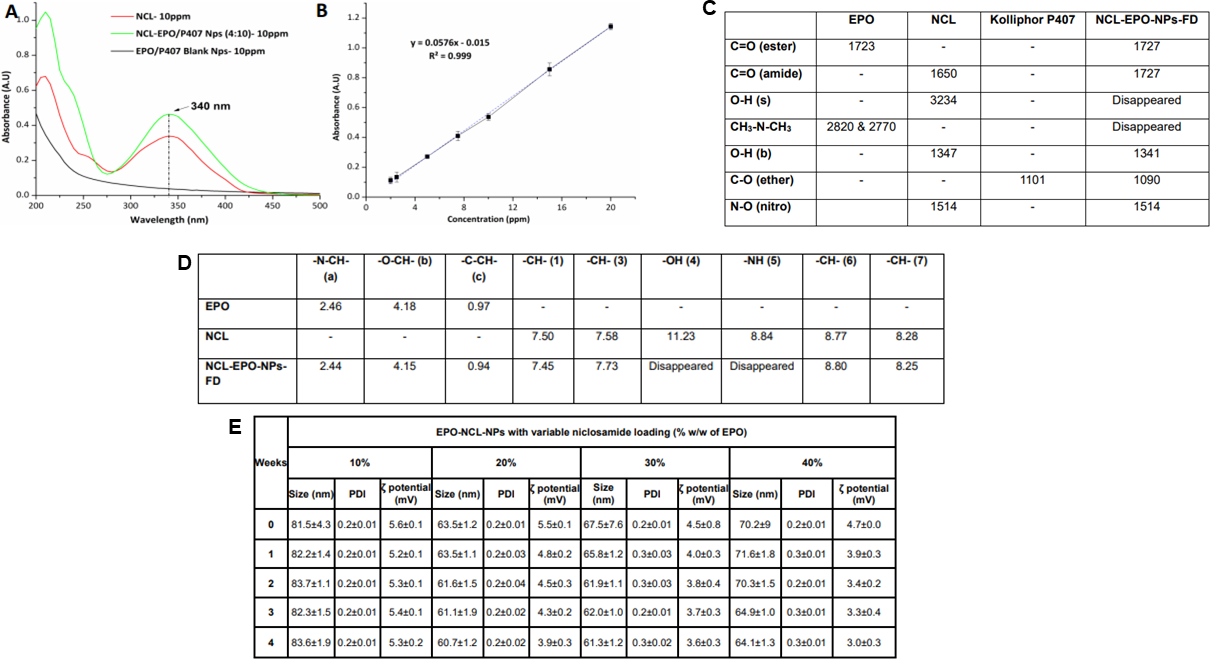

Supplement: S1 Fig — UV-Visible spectra of NCL (NCL-10 ppm), NCL-EPO NPs (equivalent to 10 ppm NCL), and EPO blank NPs (A). Calibration curve for NCL using UV-Vis Spectroscopy (n = 3 ± SD) (B). Summary of the characteristic FT-IR peaks representing the key functional groups in Eudragit EPO, NCL, Kolliphor P407, and NCL-EPO-NPs (freeze dried) (C). 1H NMR chemical shifts observed in freeze-dried NCL-EPO-NPs in comparison with EPO and NCL (D). Evaluation of particle size, PDI, and zeta potential of NCL-EPO-NPs containing different loading of NCL (% w/w EPO) on long-term storage at room temperature. (Data expressed as mean ± SD; n = 3) (E). Raw data are found in the file S1 Data. FT-IR, Fourier-transform infrared; NCL, niclosamide; NCL-EPO-NP, NCL-loaded EPO NP; NP, nanoparticle; PDI, polydispersity index. (TIF) [file pbio.3001762.s002.TIF]

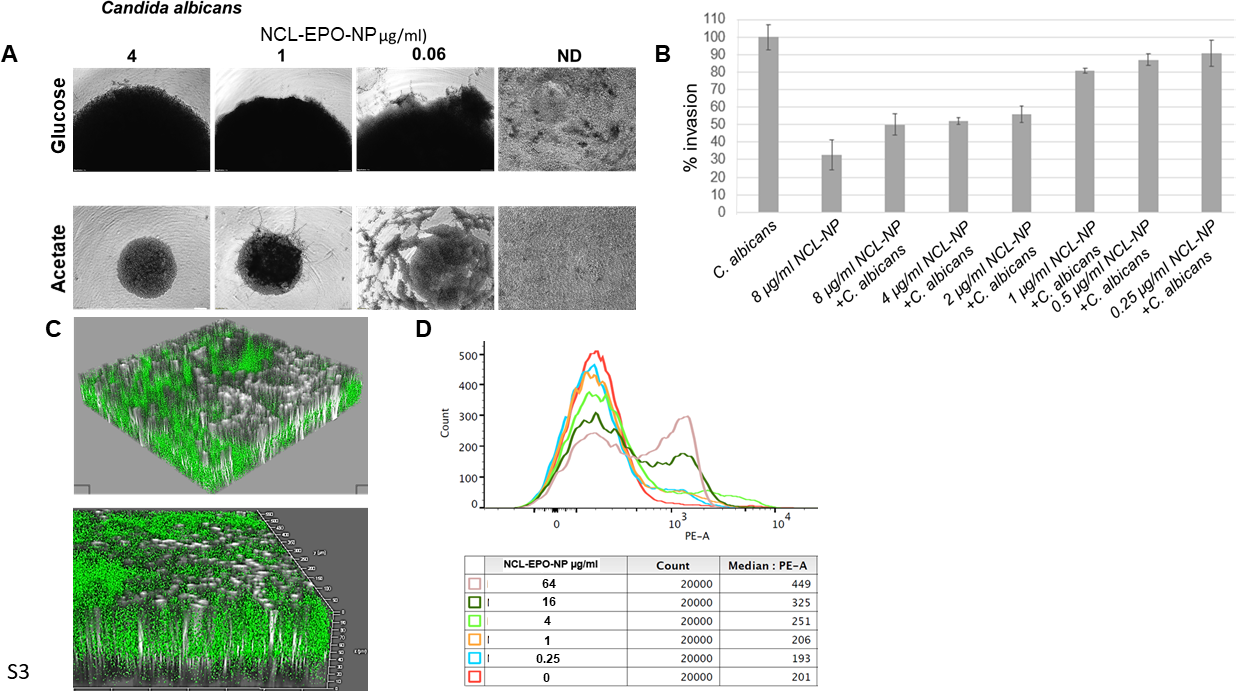

Supplement: S2 Fig — NCL-EPO-NPs prevent planktonic C. albicans growth in acetate (A), protect HUVEC from damage (B), penetrate C. albicans biofilms (C), and increase ROS activity in the fungus (D). Raw data are found in the file S1 Data. HUVEC, human umbilical vascular endothelial cell; NCL-EPO-NP, NCL-loaded EPO NP; ROS, reactive oxygen species. (TIF) [file pbio.3001762.s003.TIF]

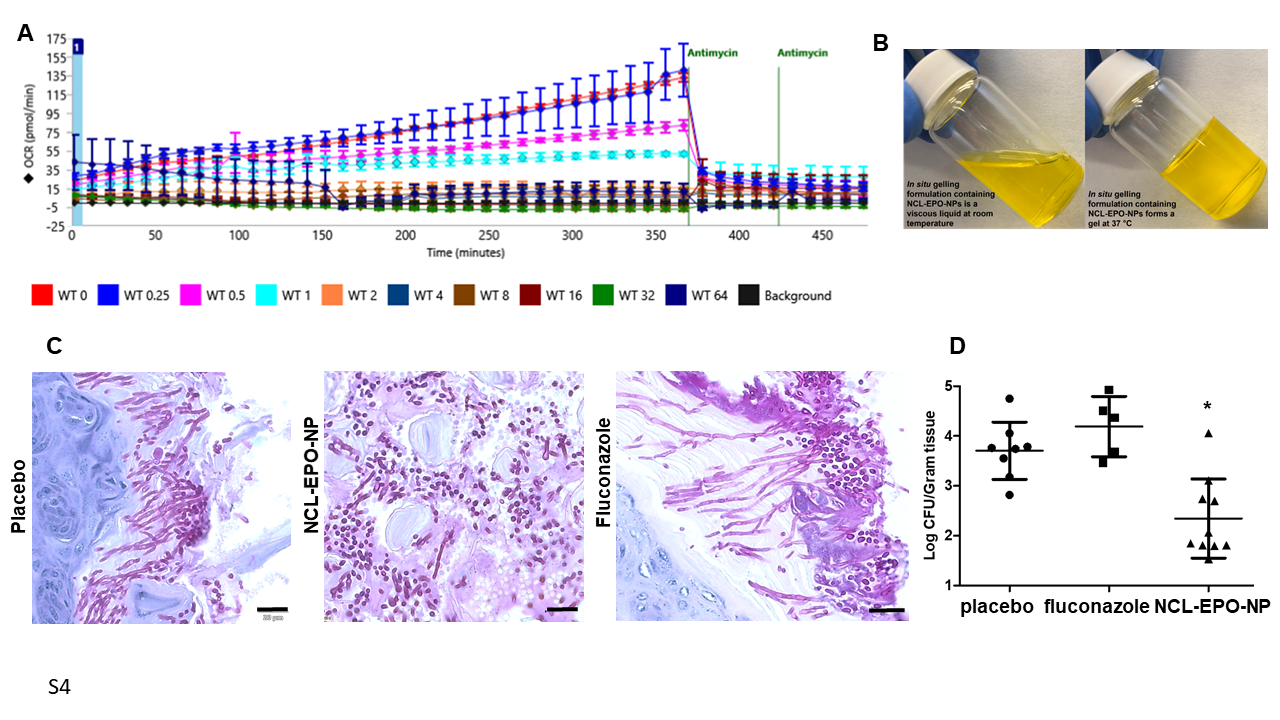

Supplement: S3 Fig — NCL-EPO-NPs reduce oxygen consumption rate in C. albicans—a time course analysis (A). NCL-EPO-NPs can be formulated as gel (B). Histology pictures from the OPC model, 40X magnification, scale = 20 μm (C). Fungal burden in vagina after drug treatment (D). Raw data are found in the file S1 Data. NCL-EPO-NP, NCL-loaded EPO NP; OPC, oropharyngeal candidiasis. (TIF) [file pbio.3001762.s004.TIF]

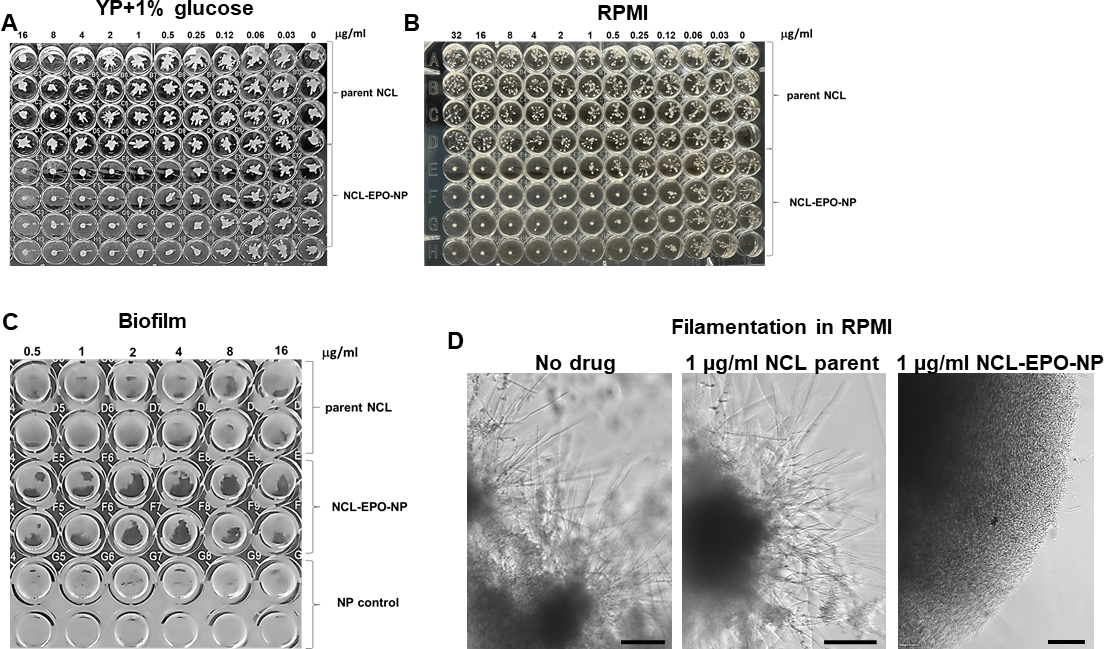

Supplement: S4 Fig — NCL parent is ineffective even at >32 μg/ml (A,B). Parent NCL or NP control did not detach biofilms, while NCL-EPO-NP detached preformed biofilms at concentrations as low as 0.5–1 μg/ml (C). NCL-EPO-NP abrogates filamentation at 1 μg/ml, while parent NCL does not, and filaments as efficiently as the no drug control. NCL, niclosamide; NCL-EPO-NP, NCL-loaded EPO NP; NP, nanoparticle. (TIF) [file pbio.3001762.s005.TIF]
